# Retraction: Enhanced heart disease diagnosis and management: A multi-phase framework leveraging deep learning and personalized nutrition

**DOI:** 10.1371/journal.pone.0351485

**Published:** 2026-06-12

**Authors:** The PLOS One Editors

The *PLOS One* Editors retract this article [1] due to concerns about:

- Compliance with PLOS policy on Manipulation of the Publication Process;
- Several references do not appear to support the statements for which they were cited, including References 3, 13-15, and others;
- Text overlap between the Results and discussion sections of [1] and [2];
- Figs 5-9 of [1] are highly similar to Figs 5-9 of [2].

Author Sandeep Dalal (SaDalal) informed PLOS that they were unaware of the article [1] and did not agree to be on the author list. SaDalal stated that they do not own the email address provided for them at the time of submission.

This article [1] was republished on May 27, 2026, to remove SaDalal’s email address and Corresponding Author designation. Please download the article again to view the correct version.

SuDalal, MD, and AH agreed with the retraction. RR, RSC, and SaDalal responded but expressed neither agreement nor disagreement with the editorial decision. IM either did not respond directly or could not be reached.

Figs 5-9 in [1] appear similar to content previously published in [2] under a CC BY 4.0 license. These figures are subject to the license that applies to the original article.
